# Occupational exposure and its mechanistic link to allergic asthma and lung function decline; a data-driven approach coupled to mining of adverse outcome pathway signatures

**DOI:** 10.3389/ftox.2025.1589380

**Published:** 2025-09-08

**Authors:** Rob Stierum, Manosij Ghosh, Marjolein Meijerink, Xavier Pinho, Joost Westerhout, Vivi Schlünssen, Anjoeka Pronk, Jolanda van Bilsen

**Affiliations:** ^1^ TNO, Risk Analysis for Prevention, Innovation and Development, Zeist, Netherlands; ^2^ KU Leuven, Environment and Health, Leuven, Belgium; ^3^ Department of Public Health – Research unit for Environment, Occupation and Health, Danish Ramazzini Centre, Aarhus University, Aarhus, Denmark

**Keywords:** sensitisers, ultrafine particles, in vitro cells, toxicogenomics, AOP, dosimetry, asthma, lung function decline

## Abstract

Within occupational epidemiology, the establishment of associations between chemical exposures and health outcome, in particular of individual chemicals present in the exposome, is difficult. Epidemiological studies are valuable but may be prone to confounders, or lack detailed exposure characterisation. Rodent studies may suffer from interspecies differences in comparison to humans. Here, we explore if a data driven approach can leverage human relevant mechanistic information to inform presumed associations between chemical exposures and two common respiratory disorders: lung function decline (LFD) and allergic asthma (AA). Using public toxicogenomics datasets, we identified Gene Ontology Bioprocesses (GO BPs) enriched in human respiratory cells, exposed *in vitro* to either diesel ultrafine particles (UFP) or respiratory sensitisers. In addition, for LFD and AA, GO BPs were curated from Molecular Initiating Events (MIEs) and Key Events (KEs) extracted from the Adverse Outcome Pathway (AOP) Wiki, and DisGeNET, a gene-disease database. Considering the commonality in GO BPs, a clear overlap was observed between GO BPs derived from UFP and LFD (a.o. “negative -“/”positive” regulation of cell activation,” “positive regulation of ion transport,” “stem cell proliferation”). 20 GO BPs were overlapping between sensitisers in combination with AA (e.g., “responses to xenobiotic stimulus,” “response to oxidative stress” and “regulation of response to cytokine stimulus”). For AA, sensitiser concentrations used in in vitro were generally higher compared to equivalent concentrations expected *in vivo* (from PBK modelling). Yet, the overlapping GO BPs discovered for these endpoints were plausible and aided in providing mechanistic insights. Currently, limitations exist in the approach to infer causality (e.g., data availability, coverage of AOPs, *in vitro* to *in vivo* dosimetry issues), however it can inform on the identification of chemicals within the occupational exposome and putative mechanistic linkage with occupational diseases. Finally, the annotated MIEs and KEs for LFD and AA may serve as valuable resource for further AOP developments.

## Introduction

Over the past years, significant improvements have been made in chemical risk assessment following advancements in toxicology. Similarly, molecular medicine, molecular epidemiology and research on the exposome, defined as the “life-course environmental exposures, from the prenatal period onwards” (Wild, 2005) have further matured, contributing to a better understanding of exposure related diseases. Yet, associations between exposure and health outcome, in particular of individual chemicals, and interactions between these within the occupational exposome (Pronk et al., 2022), in relation to health, are difficult to comprehend in humans. Responses observed in animal studies are informative but may not always be representative for humans, as these are often performed in inbred strains (ingnoring interindividual differences seen in humans), which may alter outcomes compared to studies performed in wild type animals (Brown et al., 2011). Epidemiological studies, valuable to find associations between disease and exposures may be prone to bias, confounders and uncertainties in quantitative exposure assessment in relation to health outcome (Schaefer et al., 2025).

Given these challenges, we here explore if a human-centric, data-driven approach can provide mechanistic insights to support possible associations between exposure and health effects with relevance for humans. Further, we address if this approach is suitable to identify if different chemicals within the occupational exposome induce similar mechanisms leading to adverse outcomes, as this may ultimately help to prioritise chemicals for risk assessment.

Exposure to occupational agents contributes substantially to the burden of respiratory diseases (Driscoll et al., 2005; Blanc et al., 2019). For this reason, and as part of the European H2020 Exposome Project for Health and Occupational Research (EPHOR)^1^, (Pronk et al., 2022), the effects of respirable chemicals on lung function and allergic asthma (AA), triggered by sensitisation, are considered here. Chronic obstructive pulmonary disease (COPD) and asthma (including AA, induced by respiratory sensitisers), are the most common chronic respiratory diseases globally. Recent estimates suggest that both asthma and COPD affect millions of people world wide. In 2019, asthma affected 262.4 million people globally (Wang et al., 2023), while COPD affected 212.3 million people (Safiri et al., 2022). These are chronic inflammatory diseases characterised by airway obstruction and accelerated decline in lung function with complex gene-environment interactions. Respiratory sensitisation, where the immune system becomes hyper-responsive to a substance, does not always cause immediate symptoms. However, once sensitised, subsequent exposures can trigger an immune response, potentially leading to health issues like allergic asthma (AA) (Hargitai et al., 2024). There are considerable similarities in the immune mechanism and other biological processes causing these diseases. However, the sequential activation of these is not fully understood in humans, in particular in relation to occupational exposures.

Therefore, we developed an expert- and data-driven workflow grounded in human biology to facilitate the exploration of potential links between respiratory exposures and lung diseases. This, to ultimately support epidemiological and rodent studies. To this end, we evaluated if known occupational respiratory irritants (ultrafine particles (UFP) and sensitisers (phthalic anhydride (PA), glutaraldehyde (GLUT), 2,4-Diisocyanato-1-methylbenzene (TDI), maleic anhydride (MA), trimellitic anhydride (TMA)), commonly present within the occupational exposome, causes the (transcriptional) activation of specific Gene Ontology Bioprocesses (GO BPs) (“the larger processes or ‘biological programs’ accomplished by the concerted action of multiple molecular activities” (Ashburner et al., 2000; Gene et al., 2023), that are associated with lung function decline (LFD) and AA.

Thus, the assumption was that possible mechanistic connectivity between exposure and health outcome, may be informed via GO BPs common to both model exposures and disorders. We used expert knowledge to retrieve information on Adverse Outcome Pathways (AOPs) -a concept originally from ecotoxicology (Ankley et al., 2010) - relevant to AA and LFD. We identified the relevant GO BPs, by mapping these onto the different Molecular Initiating Events (MIEs) and Key Events (KEs) within these prioritised AOPs. Additional GO BPs derived from a gene-disease centered database (DisGeNet^2^) (Bauer-Mehren et al., 2010) were also included. We then retrieved data from *in vitro* toxicogenomics studies employing different cell lines in combination with controlled chemical exposures (respiratory sensitisers, diesel exhaust) and performed GO BP enrichment analysis. Subsequent overlap analysis indeed identified relevant common GO BPs shared by the exposures and disorders, which may inform hypotheses about how specific occupational exposures link to respiratory diseases. While the approach is novel and interesting from a human-centric risk assessment perspective, at present it is likely influenced by a.o. the availability of relevant high quality human (*in vitro*) exposure-effect data, the extent to which AOPs are curated, intrinsic limitations of *in vitro* models such as the absence of interplay between different immune cells and uncertainties and issues related to *in vitro* to *in vivo* dosimetry comparisons to address the value for ultimate hazard characterisation.

## Methods

### Retrieval of GO biological processes, general considerations and steps

At the time of our analysis OECD-endorsed AOPs for LFD and AA were absent. Therefore, we relied on a well-structured expert curation process. This involved six domain experts, with expertise in respiratory clinical epidemiology, immune toxicology, respiratory toxicology, molecular toxicology and exposure sciences, who reviewed candidate AOPs and associated GO BPs through a series of documented discussions and consensus-building sessions. Comments were recorded in a shared Excel file, and decisions were finalized during multiple online TEAMS meetings. To avoid as much as possible any bias in selecting AOPs/GO BPs, the following clear inclusion and exclusion criteria guided the selection of AOPs, MIEs, KEs, and GO BPs:

1. Source Credibility: Only information from trusted and peer-reviewed sources (e.g., AOP-Wiki, PubMed, GeneCards) was considered.
2. Human Relevance: Preference was given to pathways with human gene/protein orthologues to minimize interspecies extrapolation issues.
3. Mechanistic Plausibility: Pathways were evaluated for their potential to inform on possible relationships between exposure, MIE/KE activation, and adverse outcomes.
4. Endpoint Specificity: Mechanisms deemed too generic or those exclusively describing unrelated endpoints (e.g., lung cancer development) were excluded unless directly relevant to respiratory sensitization or asthma.
5. Feedback Mechanisms: The absence of feedback loops in some AOPs was noted, and the need to consider such mechanisms in future iterations was acknowledged.
6. Cross-Domain Learning: Concepts from more mature AOP domains (e.g., food allergy) were considered where applicable. For example, mechanisms from food allergy AOPs were evaluated for their relevance to respiratory sensitization.
7. Consensus-Based Selection: Final inclusion decisions were made by consensus among the expert panel, ensuring that selected pathways were both biologically plausible and contextually relevant.

To complement the expert curated GO BPs, we also used DisGeNET. DisGeNET contains, aside the inclusion of information from expert curated databases, also information retrieved in an unbiased manner from textmining of published literature.

### Retrieval of GO biological processes related to LFD

**FIGURE 1:**
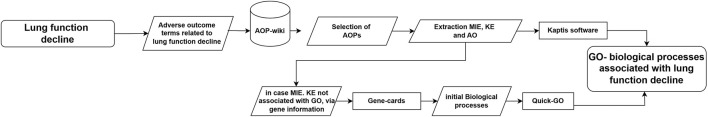
Workflow employed to derive GO BPs of relevance to LFD. AOP resources (AOP Wiki, Wiki Kaptis) were searched and combined with expert knowledge to obtain a set of GO BPs, for inclusion in subsequent overlap comparison with GO BPs obtained from toxicogenomics data.

Using the criteria outlined above, an extensive expert search was conducted in AOPwiki^3^, for AOPs of possible relevance to LFD, including information on asthma and COPD. A search was performed with the following terms “LF: lung function; RS: Respiratory symptoms; AS: Asthma; COPD)” followed by additional search terms: “Cough, fibrosis, lung cancer”. After this, AOPs were further selected in an expert based manner. Next, based on evaluation of these AOPs, the MIE, KE and outcomes were extracted by experts. In order to enable the subsequent integration of GO BP based on enriched gene sets from *in vitro* toxicogenomic data (described below) with adverse outcome pathway content, the MIEs and KEs derived were mapped onto GO terms. This was done by using Wiki Kaptis^4^, for retrieval of GO terms associated with the MIE/KE. In cases only gene names were specified, without a direct link of the MIE/KE to GO terms (e.g., “Event ID: 1911; FOXJ1 Protein, Decreased”) Gene Ontology information was retrieved from GeneCards^5^. Next, this information was used to extract the primary GO and child GO terms from the QuickGo database^6^. Figure 1 summarises the steps taken to derive GO BPs for LFD, and asthma.

### Retrieval of GO biological processes related to AA

**FIGURE 2:**
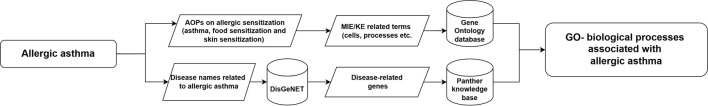
Workflow employed to derive GO biological processes with relevance to AA. AOP and literature resources (AOP Wiki, Pubmed) and disease-gene information sources (DisGeNet) were searched to obtain a set of GO biological processes, for inclusion in subsequent comparison with GO BPs obtained from toxicogenomics data.

In order to find mechanisms for AA, using the criteria outlined above, we queried various sources (Figure 2). First, similar as done for LFD, existing AOPs (including the ones in development) related to allergic lung sensitisation were explored to identify relevant MIE and KE. For this, the AOPwiki^3^ and Pubmed^7^ were searched for relevant AOPs. The retrieved information related to sensitisation in the lungs was considered not complete enough, as only parts of the AOP-cascade were described, e.g., the AOP 39^8^ describes only covalent protein binding, “covalent binding to proteins leads to Respiratory Sensitisation/Sensitization/Allergy”). Therefore, AOP information on food sensitisation described in Van Bilsen et al. (2017) and the corresponding AOP on “Sensitization induction of the intestinal tract by food proteins”^9^, as well as the AOP for skin sensitisation^10^, were considered to fill in gaps in the understanding how sensitisers may affect cells, cell-cell interactions and tissue homeostasis, ultimately leading to AA.

From these resources, relevant MIE/KE were selected, and the GO database^11^ was searched to identify relevant biological processes (GO BPs) related to MIEs/KEs of possible relevance of AA. Secondly, as AOPs are linear and unidirectional, they do not contain compensatory mechanisms and are limited to the sensitisation phase of asthma and not the elicitation phase (including the roles of mast cells, basophils, eosinophils, etc.), the retrieved information of the AOPs as described above was considered too limited (as also shown in the results section). Therefore, the DisGeNET database^2^ was accessed to identify additional genes associated with AA. These retrieved genes were used as input into a Panther overrepresentation analysis^12^ to identify possibly relevant GO biological terms. After retrieving the GO BP from these two different sources, they were merged together and duplicates were removed. Figure 2 summarises the workflow followed.

### Selection of relevant chemicals and analysis of associated transcriptomics data for LFD and AA

We followed a structured and predefined approach to identify suitable chemicals and toxicogenomics studies/datasets. The selection process was guided by the following “overall criteria” steps:

1. Chemical Relevance: well, unequivocally established exposures causing lung function decline, as well as sensitisation. This based upon literature evidence, expert knowledge and ECHA resources.
2. Comprehensive Data Retrieval: we searched multiple databases (Comparative Toxicogenomics Database (CTD), biostudies.org, in particular the diXa data warehouse^13^, as this contains the legacy of toxicogenomics data from several past EU research projects; Pubmed^7^; ToxicoDB^14^, a database containing multiple large toxicogenomics datasets; and gene expression omnibus^15^ to retrieve relevant gene and omics datasets and metadata, associated with these chemicals;
3. Human-Relevant Models: we prioritized datasets derived from human *in vivo* studies (none were available) or human organ-specific *in vitro* systems (e.g., lung epithelial cells, dendritic cells) representative for (parts of) the organs/tissues/cells suspected to be involved as the site of MIE/KE/AOPs, to avoid interspecies extrapolation issues;
4. Genome-Wide Expression Coverage: only studies with full-genome expression data were considered, enabling robust GO Biological Process (GO BP) enrichment and overlap analysis;
5. Data Quality and Accessibility: we required datasets to include clearly defined chemical-specific differential gene expression, (as identified and described by the authors) as well as access to annotated raw data files;
6. Mechanistic Breadth: to ensure mechanistic robustness and avoid spurious findings (e.g., specific to one chemical only), we further narrowed our selection to studies in which the multiple set of same chemicals were tested across different *in vitro* models. This allowed us to explore consistent gene expression responses across cell types, for multiple sensitisers, and better capture molecular initiating events (MIEs) and key events (KEs) relevant to allergic asthma (AA) and lung function decline (LFD).

Below, the selection process and downstream data analysis is described in more detail for LFD and AA. Concerning LFD, the selection was confined to studies involving diesel motor emission, including ultrafine particles (UFP), as this exposure has been clearly associated with LFD in humans (Zhang et al., 2017; Du et al., 2020). Diesel exposure is amongst the most common exposures within the occupational exposome (Peters et al., 2024). Therefore, the assumption was that the mechanistic connectivity via overlap in GO BPs, could be potentially observed in this model. Following the stringent criteria outlined above under “overall criteria”: one study, in compliance with all the criteria outlined above, on ultra fine particles from diesel combustion was ultimately retrieved (Grilli et al., 2018) (see Results for details). This study employed RNAseq analysis, and the data were retrieved from ArrayExpress under BioStudies via accession number E-MTAB-5157^16^. Normalisation of the RNAseq data was carried out with edgeR package (version 4.0.12). Differential gene expression analysis was confirmed by comparing gene expression profiles obtained from samples treated with UFP generated from diesel, with time matched untreated control exposures. For each time point, gene expression was used as input into GO BP enrichment analysis. To this end, the gene lists along with their respective fold changes were subsequently subjected to a Gene Set Enrichment Analysis (GSEA) through the web-based tool WebGestalt^17^. The chosen enrichment methodology involved GSEA for organism *Homo sapiens*, utilizing the Gene Ontology–Biological Processes functional database. Biological processes with a False Discovery Rate (FDR) below 0.25 were selected for further analysis.

Sensitizing chemicals were identified in line with the stringent “overall criteria” (step 1, above) to ensure the retrieval of high quality studies fit for the purpose of our study. In detail, first, expert knowledge was employed (respiratory clinical epidemiology, immune-, respiratory- and molecular toxicology, and exposure sciences), taking into consideration the following publications (Baur and Bakehe, 2014; Clausen et al., 2020; Dalbøge, Albert Kolstad et al., 2022; Moual et al., 2016). Additionally, a recent overview publication on respiratory sensitisers for application in new approach methodologies (*in silico*, *in vitro* cell based assays) (Sadekar et al., 2022) was used. Finally, the ECHA website^18^ was searched for “respiratory sensitiser” under the “harmonised classification and labelling” to verify if the chemicals selected were indeed also classified as respiratory sensitiser in humans. The retrieval of studies with omics datasets for identified chemicals was as described above under “overall criteria.” Applying the stringent criteria, two studies (Dik et al., 2015; Forreryd et al., 2015) were found, in which 5 relevant chemicals were studied (more details see Results).

Data from the Dik et al. (2015) were kindly shared by the authors upon personal communication. For these datasets, bioinformatics analyses were conducted in R-4.3.1^19^ using the open software Bioconductor (version 3.18). Quality control and Normalisation of the Affymetrix CEL files were performed using the Robust Multichip Average (RMA) method, from the affy package (version 1.78.2). The microarray raw data (.CEL files) from Forreryd et al. (2015) was obtained from ArrayExpress (under BioStudies) wit accession number E-MEXP-3773^20^. Gene expression changes were compared for each of the chemicals *versus* the correspondent control (e.g., Phthalic Anhydride (PA) *versus* DMSO). Subsequent GO BP enrichment analysis was performed as described above for LFD.

### Overlap analysis between GO BPs from diesel UFP and sensitisers, and GO BPs associated with AOPs relevant for LFD and AA

For LFD, overlap analysis was performed to identify common GO BP enriched in the toxicogenomics data and derived by AOP data mining/expert review as relevant to LFD. For each overlap analysis, a Venn Diagram was generated and a Jaccard Index was calculated, according to formula: J(A,B) = ∣A∩B∣/∣A∪B∣. The Jaccard index is a commonly used measure of the similarity between two sets. It is defined as the size of the intersection divided by the size of the union of the sample sets. This index gives a measure between 0 and 1, where 0 means no overlap and 1 means complete overlap, so the higher the index, the larger the overlap is. The Jaccard index is normalised to account for differences in set sizes, allowing us to compare sets independently. The Jaccard Index Analysis is here employed to compare different scenarios (exposure, disease) with one another in a standardized way across different chemicals. The Jaccard index is suitable to balance out difference in the size of each of the GO BPs sets, so the outcome of this analysis does not get penalized (influenced) by the number of GO BPs in each of the sets of GO BPs.

For the 5 respiratory sensitisers tested in the two different *in vitro* systems (Dik et al., 2015; Forreryd et al., 2015), the GO BP overlap analysis was performed in a similar manner, with the notion that expert curated GO BPs relevant to AA were used. An expert evaluation by an immunologist and toxicologist was performed to verify the plausibility of the proposed exposure-to-health effect relations from a mechanistic perspective. This interpretation focused on a limited number of exposures, specifically ultrafine particles (UFP) from diesel exhaust and phthalic anhydride (PA), in relation to GO BPs for LFD and AA.

### Exploration of *in vitro* dosimetry employed in relation to equivalent internal concentrations *in vivo*, expected from external exposure scenarios

Next, we explored if the concentrations of chemicals used in the identified *in vitro* studies were relevant in comparison to internal concentrations, resulting from external exposures scenarios. We limited this exploration to sensitisers only, as diesel UFP is a complex mixture composed of many different chemical constituents, e.g., organic compounds, organic and elemental carbon, alkanes, metals (e.g., Pb, As, Cd) and PAHs, for which *in vitro* to *in vivo* comparison of dosimetry of individual constituents is too complicated (Lim et al., 2021).

To this end, the concentrations employed in Dik et al. (2015), Forreryd et al. (2015) were compared to expected internal concentrations predicted/measured in relation to external exposure scenarios. Either physiologically based kinetic (PBK) modelling, biomonitoring data and/or combinations thereof, can serve this purpose. PubMed queries were performed in which the name of the chemical was combined with “PBK model,” “internal dose/concentration,” “biomonitoring,” “biomarkers of exposure” in title/abstract. For TDI, we modified a PBK previously developed by our group (Scholten et al., 2023). In addition, Microsoft 365 Copilot Chat was used with the following prompt: “For the following 5 chemicals I would like to have all information available from scientific literature that can provide me an answer as to the relation between quantitative external exposure and quantitative internal exposure, ideally within the lung. You can consider published PBK/PBPK models, as well as biomonitoring data in direct relation to quantitative external exposure information. I do not want to perform any PBK modelling myself. Here are the five chemicals: phthalic anhydride, glutaraldehyde, 2,4-Diisocyanato-1-methylbenzene, maleic anhydride, trimellitic anhydride.” The results were further evaluated by exploration of the original scientific sources underlying the Copilot statements.

## Results

### Retrieval of biological processes associated with LFD and AA

**FIGURE 3:**
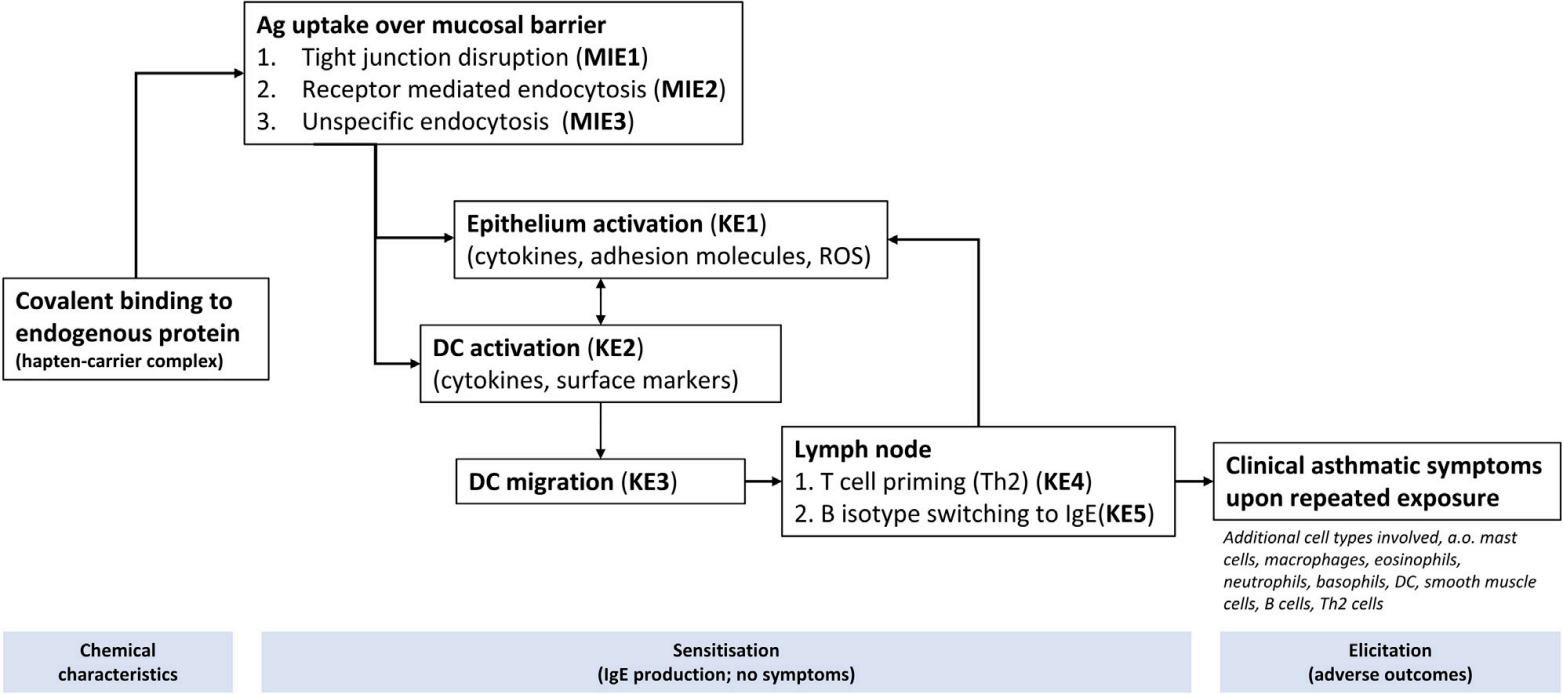
Key mechanisms (depicted as KE, MIE) assumed to be associated with AA induced by occupational exposures, based on AOP 39 (https://aopwiki.org/aops/39 and published AOPs from food sensitisation and skin sensitisation. Figure adapted from Van Bilsen et al. (2017).

For LFD, a list of AOPs was retrieved from AOP-Wiki^3^, and mapped to their respective GO BPs. A total of 9 AOPs were retrieved (AOP 418, AOP 419, AOP 148, AOP 302, AOP 411, AOP 424, AOP 425, AOP 39, AOP 196), related to LFD including asthma and COPD (Supplementary Data File S1, sheet tab “list of AOPs,” in relation to Figure 1 workflow step “selection of AOPs”). It is important to mention that most of the retrieved AOPs were in early stages of development, with very few ‘Open for comment’. From the AOPs, a list of unique molecular initiating events (MIE) and Key events (KE) were summarised and were mapped to their primary GO and child GO terms from the QuickGO database. These are available in Supplementary Data File S1, sheet tab “AOP event to QUICK GO”, in relation to Figure 1 workflow step “Quick-GO”. From this, a total of 29 parent GO term and 185 linked child GO terms were retrieved and after removing duplicates 166 GO terms were retained. These are available within Supplementary Data File S1, sheet tab “Final list of GO”, in relation to Figure 1 workflow step “GO-biological processes associated with lung function decline”. Following a similar approach for AA, Figure 3 shows several putative MIEs/KEs derived from expert curation of events published in AOPs on food and skin sensitisation. A textual hypothetical description of these results is the following. Allergic asthma is a complex disorder involving several key events that lead to clinical symptoms. Chemicals that induce allergic asthma can undergo covalent binding to endogenous protein, known as haptenisation, either before (binding to extracellular proteins in the airway lining fluid) or after (binding to intracellular protein or cytosolic peptides) crossing the mucosal barrier in the lungs, depending on their reactivity and solubility. The formed complex can be recognised by the immune system. The sensitisation process begins with allergens when allergens interact with the airway epithelium, activating it. Allergens, particularly those with proteolytic activity like house dust mite proteins, can disrupt tight junctions between epithelial cells, compromising the mucosal barrier integrity and allowing allergens to penetrate the epithelium. Allergens can also cross the mucosal barrier through receptor-mediated endocytosis and unspecific transcellular transport. This crossing triggers epithelial activation, leading to the release of pro-inflammatory mediators that attract dendritic cells (DCs) to process the allergens. The activated DCs then migrate to lymph nodes, where they prime naive T cells, promoting their differentiation into Th2 cells. Simultaneously, B cells undergo isotype switching to produce allergen-specific IgE antibodies. Upon re-exposure to the allergen, cross-linking of IgE on mast cells results in the release of inflammatory mediators, causing bronchial smooth muscle contraction, increased mucus production, and airway inflammation. These events collectively manifest as the clinical symptoms of allergic asthma, including wheezing, coughing, and difficulty breathing.

Table 1 provides an overview of the final number of GO BPs retrieved for AA, in relation to the MIE/KEs that were considered relevant (Figure 3). The number of GO BPs curated was rather modest and the information derived from AOP and GO resources may be too limited to capture the full mechanism, as this mainly covers the sensitisation phase (and not the elicitation) and does not include feedback loops. Therefore, to retrieve additional GO BPs possibly associated with AA caused by occupational sensitisers, DisGeNet followed by PANTHER Overrepresentation Test were used to retrieve the additional genes and GO BPs, respectively, associated with these endpoints. The outcome of this analysis is shown in the far right column of Table 1. Interestingly, a much larger number of GO BPs (2205) was observed compared to the GO BPs derived from expert curated KE/MIEs. This number represents ∼8.1% of all GO BPs, suggesting that a substantial amount of GO BP are associated somehow to AA (2205/27047 Biological process terms (27047: based upon statistics for release 2014-04^21^.

**TABLE 1:** Overvi ew of the number of selected GO BP associated with key events of AA. The left columns represent the number of GO BPs collected from an expert based curation of AOP and GO resources in relation to a key mechanisms of AA (Figure 3). Far right column indicates the number of GO BPs derived from DisGeNet. The GO BPs with GO Term ID and Go Term Name, are shown in detail in Supplementary Data File S2.

	GO BPs associated with key events of AA										DisGeNet
AOP step	Tight junction^a^	Endocytosis^a^	Sampling^a^	Dendritic cell activation^b^	Dendritic cell migration^b^	T cell priming	Isotype switching	B cell activation	Type 2 immune response^ab^	Hypersensitivity^ab^	DisGeNet AA^ab^
Numbering in cascade (See Figure 3)	MIE1	MIE2-3	MIE2-3 & KE1-2	KE2-3	KE3	KE4	KE5	KE5	All	All	All
#GO BPs selected to be present	17	14	5	11	6	18	11	43	13	6	2205

^a,b^Different sets of GO BPs, were used in subsequent GO BP, overlap analyses with toxicogenomics data from two *in vitro* studies.

^a^ GO BPs, used with data from Dik et al. (2015).

^b^ GO BP, used with data from Forreryd et al. (2015). The decision to include different sets of GO BPs, for overlap analysis was based upon the expected presence or absence of MIEs/KEs, in relation to the characteristics of the *in vitro* models. As none of the two models was expected to address T-cell priming, Isotype switching or B cell activation, these were omitted from subsequent downstream GO BP, overlap analysis.

Supplementary Data File S2 provides in each of the tabs the different expert and literature derived MIEs/KEs, also reflected in Figure 3. Each tab contains the final set of GO BPs terms selected that fit each of the different MIEs/KEs possibly involved in an AOP for AA induced by occupational exposures.

### Selection of relevant chemicals and toxicogenomics datasets in relation to LFD and AA

For selection of studies/datasets in relation to relevant chemicals, selection criteria were applied (see Methods, “overall criteria”). Considering LFD, one study was identified (Grilli et al., 2018), in which human bronchial epithelial cells (BEAS-2B) were exposed *in vitro* to ultrafine particles (UFP) derived from diesel (and biomass) combustion. Cells were treated with 2.5 μg/cm^2^ (equivalent to 25 μg of UFP particles/mL of tissue culture media), and RNA was collected at 1, 4, 8, 16, and 20 h, subjected to RNA seq analysis (for data see Biostudies under accession number E-MTAB-5157^18^, and compared to time matched control exposures.

Concerning AA, following, two toxicogenomics studies (Dik et al., 2015; Forreryd et al., 2015) were found (Table 2). Dik et al. (2015) used the human bronchial epithelial cell line 16HBE14o, an *in vitro* model representing the barrier function of the airway epithelium, in combination with Affymetrix GeneChip™ HT HG-U133+PM, with test chemical concentrations between 150 and 2730 μM. The study by Forreryd et al. (2015) used the human MUTZ-3 cell line^22^ representing a dendritic cell model. Chemicals were tested in a concentration range of 10–500 μM. These studies tested five common respiratory sensitizers in a consistent manner to provide potential insights into MIEs, KEs involved in epithelial and initial immune cell responses in relation to the GO BPs curated in relation to AA (also reflected in Table 1 with “^a,b^”). Whereas the Dik et al. study used 4 h exposure at concentration 80% cell viability, Forreryd et al., performed exposures for 24 h but did not specify a specific rationale for selecting concentrations. Interestingly, the concentrations of all chemicals employed by Forreryd et al. was around one order of magnitude lower (∼3.8–18.2 fold), compared to Dik et al. (Table 2). Both datasets were processed using the same bioinformatics pipeline (R 4.3.1, Bioconductor 3.18) (Du et al., 2008), ensuring analytical consistency.

**TABLE 2:** Selection of relevant chemicals and studies for collection of exposure related GO BPs. Estimated internal concentrations, resulting from external exposure scenarios, using PBK and/or biomonitoring data are provided.

Chemical	Abbreviation	CAS number	Pubchem link	Dik et al. (2015) 16HBE14o (Epithelial) 4h exposure at concentration 80% cell viability	Forreryd et al. (2015) MUTZ-3 (dendritic) 24h exposure, performed in biological triplicates	Estimated internal concentration at external exposure scenarios, together with methods indicated in between parentheses
phthalic anhydride	PA	85-44-9	https://pubchem.ncbi.nlm.nih.gov/compound/Phthalic-anhydride	1330 μM	200 μM	2460–114800 μM (biomonitoring: urinary concentration, (Pfaffli, 1986))
glutaraldehyde	GLUT	111-30-8	https://pubchem.ncbi.nlm.nih.gov/compound/3485	190 μM	10 μM	0.08–0.16 μM (PBK: plasma Cmax, (Cable et al., 2025)
2,4-Diisocyanato-1-methylbenzene	TDI	584-84-9	https://pubchem.ncbi.nlm.nih.gov/compound/11443	150 μM	40 μM	0.023 µM in lung Interstitial fluid 0.575*10^–3^ μM in lung epithelial lining fluid External exposure at 40 μg/m^3^ for 4 h exposure. (PBK, based upon (Scholten et al., 2023) see Supplementary Data File “S9_PBK_modelling_TDI”)
maleic anhydride	MA	24937-72-2	https://pubchem.ncbi.nlm.nih.gov/compound/Maleic-Anhydride	2650 μM	500 μM	Unknown (serum MA-specific IgE antibodies, (Hansen et al., 2014))
Trimellitic anhydride	TMA	552-30-7	https://pubchem.ncbi.nlm.nih.gov/compound/11089	2730 μM	150 μM	∼0.009–0.01 µM (low exposure) ∼0.17–0.19 µM (high exposure) (PBK: plasma Cmax (Cable et al., 2025)

The chemicals tested were phthalic anhydride (PA), glutaraldehyde (GLUT), 2,4-Diisocyanato-1-methylbenzene (TDI), maleic anhydride (MA) and trimellitic anhydride (TMA)). These chemicals are relevant as to the occupational exposome. Phthalic anhydride (PA) is a chemical intermediate in the plastics industry for various phthalate esters, which in turn are plasticisers in synthetic resins. Glutaraldehyde (GLUT) is used as a cleaning and disinfecting agent for cleaning of heat sensitive medical equipment such as endoscopes, surgical instruments, as well as for laundry and fabric treatment. 2,4-Diisocyanato-1-methylbenzene (TDI) is used in producing polyurethane products such as foams, and coatings used, for example, in bedding, furnitures and packaging. Maleic anhydride (MA) is used amongst others to form unsaturated polyester resins that can be subsequently used in construction, manufacturing of transport (boats, cars) and electrical goods, as well as in the synthesis of pesticides and other organic compounds. Trimellitic anhydride (TMA) is used in the synthesis of plasticisers for polyvinyl chloride resins, paint resins and in the nail polishing industry (as part of a PA, TMA and glycols copolymer, which was identified as an allergen (Nassif et al., 2007).

### Analysis of transcriptomics data for LFD and AA, enrichment of GO BPs and GO BP overlap analysis to associate chemical exposure to LFD and AA

**FIGURE 4:**
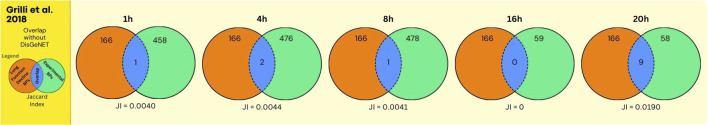
Overlap between GO BPs from LFD and diesel UFP using data from the human bronchial epithelial cells (BEAS-2B). The number of overlapping GO BPs (in blue) is shown between GO BPs enriched in a toxicogenomics study () using the bronchial epithelial cell BEAS-2B exposed *in vitro* for various durations (hours) to ultrafine particles derived from diesel exhaust (in green) and GO BPs derived from expert curation of the MIEs and KEs collected from the AOP Wiki in relation to LFD including asthma and COPD (in orange).

**FIGURE 5:**
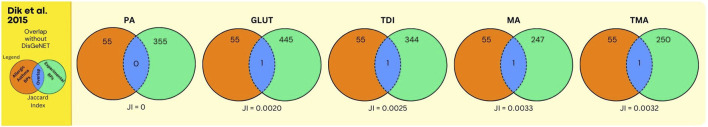
Overlap between GO BPs from AA and sensitisers using data from the human bronchial epithelial cell line 16HBE14o. The number of overlapping GO BPs (in blue) is shown between GO BPs enriched in a toxicogenomics study using the human bronchial epithelial 16HBE14o exposed *in vitro* to 5 sensitizing agents (in green) and GO BPs derived from expert annotation and mining of AOP and GO resources associated with AA (in orange).

**FIGURE 6:**
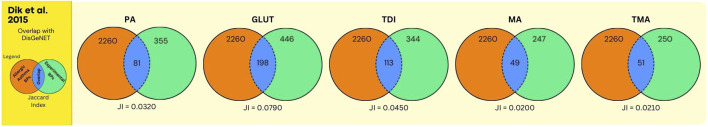
Overlap between GO BPs from AA and sensitisers using data from the human bronchial epithelial cell line 16HBE14o. The legend is further identical to the legend of Figure 5, except that here the orange circle refers to GO BPs derived from expert annotated AOP content, augmented with GO BPs derived from a gene centered approach using DisGeNet.

**FIGURE 7:**
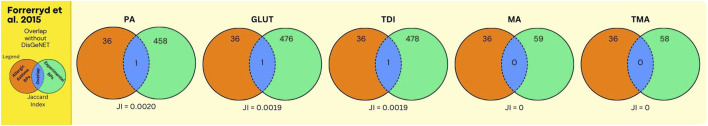
Overlap between GO BPs from AA and sensitisers using data from the human dendritic MUTZ-3 cell line. The number of overlapping GO BPs (in blue) is shown between GO BPs enriched in a toxicogenomics study (Forreryd et al., 2015) using the human dendritic MUTZ-3 cell line exposed *in vitro* to 5 sensitizing agents (in green) and GO BPs derived from expert annotation and mining of AOP and GO resources associated with AA (in orange).

**FIGURE 8:**
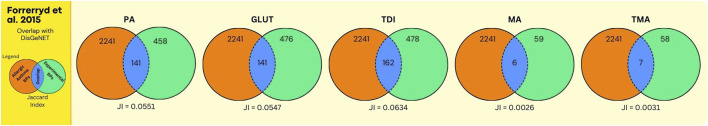
Overlap between GO BPs from AA and sensitisers using data from the human dendritic MUTZ-3 cell line. The legend is further identical to the legend of Figure 7, except that here the orange circle refers to GO BPs derived from expert annotated AOP content, augmented with GO BPs derived from a gene centered approach using DisGeNet.

For LFD (Grilli et al., 2018) the outcome of the differential gene expression analysis is shown in Supplementary Data File S3. For AA, the outcome is shown in Supplementary Data File S4, S5. In these three supplementary data files, for each of the Microsoft Excel sheet tabs, Column A represents “Gene ID”, column B represents “Fold Change Value.” These data served as input for GO BP enrichment analyses. Overlap analyses was performed between GO BPs enriched and the GO BPs relevant to LFD and AA, respectively. The outcome is shown in Figure 4 for LFD, and Figures 5–8 for AA. In more detail, Supplementary Data File S6 shows the GO BP enrichment for the Grilli et al. (2018). study, for each of the different exposure durations. Similarly, for Dik et al. (2015) and Forreryd et al. (2015), the outcome of the enrichment analysis is shown in Supplementary Data File S7,S8, respectively. In each of these files, the first excel sheet tab contains a reading guide, followed by sheet tabs representing the outcome for the different chemicals. For each of the different experimental conditions, the overlap in GO BPs is indicated in column N. For AA, this also includes the overlap derived from expert curated as well as DisGeNET derived GO BPs.

Considering the possible association between ultrafine particles derived from diesel exhaust and LFD, the overlap between experimental GO BPs and disease GO BPs is moderate (Figure 4, Supplementary Data File S6, for each exposure duration, indicated in column N of the respective sheet tabs). This, in particular at earlier exposure durations, whereas at 20 h, 9 out of 166 expert curated GO BPs were overlapping (with highest Jaccard Index: 0.0190). Despite the moderate overlap, interestingly, common GO BP were enriched across different time points: negative (GO:0050866, 1 h) or positive (GO:0050867, 20h) regulation of cell activation, positive regulation of ion transport (GO:0043270: 4 h, 20 h) and stem cell proliferation (GO:0072089: 8h,20h). This overlap in GO BPs may be informative for a mechanistic association between UFP exposure and health outcome (LFD) (see Discussion Section for interpretation).

Next, the results on chemical sensitisers, in relation to AA are shown. In Figure 5, and Supplementary Data File S7, the overlap analyses between the GO BPs corresponding to expert curated AOP content and GO BPs that were enriched in the *in vitro* experiment using human bronchial epithelial 16HBE14o cell line (Dik et al., 2015) is shown. For all chemicals, this overlap is moderate but present, with for GLUT and TDI one single GO BP (GO:0042092, “type 2 immune response”), corresponding to the expert curated MIE/KE term “T cell priming.” Similarly, for MA and TMA one single GO BP (GO:0006907, “pinocytosis”), corresponding to the expert curated MIE/KE term “endocytosis” was observed to overlap. For PA, no overlap was observed. In Figure 6 (Supplementary Data File S7), the overlap is shown, between the toxicogenomics data and expert curated GO BPs content, but now augmented with a gene centered approach using DisGeNet. As is evident, for all chemicals, the overlap was substantially larger, as higher Jaccard Indices were observed.

The analyses using data from Forreryd et al. (2015), employing a dendritic cell model, provided similar observations. Similar, as for the Dik et al. study, only one GO term “GO:0042092, ‘type 2 immune response’”, retrieved via expert curation of AOP content, was found to be enriched within toxicogenomics data for GLUT and TDI (Figure 7, Supplementary Data File S8). Apparently, the activation of this GO BP by chemical sensitisers is “conserved” throughout these different *in vitro* models. Here also, for all chemicals, higher Jaccard Indices were observed whenever the DisGeNET derived GO BPs were included in the analysis (compare Figures 7, 8). Together, these data indicate that upon expert derived curation of AOP content limited relevant GO BPs processes are found that overlap with experimental exposure related toxicogenomic data, however, the overlap between toxicogenomic and diseases data are more profound, whenever GO BP were enriched with DisGeNet derived GO BPs. Whenever this overlap is considered in more detail across these two studies, it is interesting to note that in both studies, in terms of number of GO BPs, the overlap was highest for 3 chemicals (PA, GLUT, TDI) (Figures 6, 8) in which these chemicals have the highest Jaccard Indices). This despite the fact, within each of the studies, approximately equal or much higher concentrations were used of MA and TMA, compared to PA, GLUT and TDI, respectively. Apparently, for these 5 sensitisers, these *in vitro* models are most sensitive to PA, GLUT, TDI, irrespective of the difference in *in vitro* concentration used. To explore if a data driven approach can provide mechanistic insights linking exposure to AA, as done above for UFP and LFD, we take, as an example, a closer look at the results for PA. Considering the analysis of the bronchial epithelial 16HBE14o cells (file S7_Dik_overlap_AA.xlsx; sheet tab PA; column N ‘overlap’) 81 GO PBs were overlapping. Considering the dendritic MUTZ-3 cells (file S8_Forreryd_overlap_AA.xlsx; sheet tab PA; column N ‘overlap’), 141 GO BPs were overlapping. This suggests a possible mechanistic connectivity (see Discussion Section for interpretation).

We also explored the hypothesis if the approach presented here is suitable to identify if different chemicals induce similar mechanisms leading to adverse outcomes, which may be ultimately helpful for prioritisation of chemicals for hazard assessment within the occupational exposome. To address this, we consider sensitisers, specifically the outcome of the analysis of the Dik et al. study data in further detail. Supplementary Data File S7 (sheet tab “overlapping GOs all chem”, top 101 rows) shows the GO BP terms that were found to: 1) overlap with the GO BPs associated with AA; 2) being common across all the five chemical tested (PA, GLUT, TDI, MA, TMA, chemicals indicated by different colours). 20 GO BP terms were found to be in common. In considering these in more detail, 4 GO BPs are possibly directly linked to the chemical exposure: “cellular response to external stimulus” (GO:0071496); “response to acid chemical” (GO:0001101); “response to toxic substance” (GO:0009636); “response to xenobiotic stimulus” (GO:0009410). Further, the formation of oxidative stress/reactive oxygen across all 5 chemicals species may be evidenced from: “reactive oxygen species metabolic process” (GO:0072593); “response to oxidative stress” (GO:0006979); “response to oxygen levels” (GO:0070482); “small molecule catabolic process “(GO:0044282); “regulation of small molecule metabolic process” (GO:0062012) and “fatty acid metabolic process” (GO:0006631) may be indicative for perturbations in the metabolism of molecules in general. All 5 chemicals appear to affect Immune cell responses and (intracellular signalling in relation to proinflammatory) cytokines (granulocyte activation (GO:0036230); I-kappaB kinase/NF-kappaB signaling (GO:0007249); regulation of response to cytokine stimulus (GO:0060759)), as well as some miscellaneous GO BPs (negative regulation of transport (GO:0051051); organic acid biosynthetic process (GO:0016053); protein localization to mitochondrion (GO:0070585); response to fluid shear stress (GO:0034405); response to leptin (GO:0044321); response to metal ion (GO:0010038); response to nutrient levels (GO:0031667)).

### Exploration of *in vitro* dosimetry employed in relation to equivalent internal concentrations *in vivo*, expected from external exposure scenarios

In order to explore if *in vitro* concentrations used are physiologically relevant in relation to equivalent internal concentrations expected to occur upon realistic exposure scenario, existing PBK models and/or biomonitoring data were explored. The outcome of this analysis is presented in Table 2, right column.

For PA, no published PBK models were available. Pfaffli (Pfaffli, 1986) compared external exposure to PA and internal urinary concentrations. In occupational exposure settings, urinary concentrations ranged from 0.3–14.0 μmol/mmol creatinine, collected at different times of the day. Expressed as mmol per liter of urine, these concentrations would yield estimates of 2.46–114.8 mM^23^. These values seem very high and may be overestimated, also compared to the concentrations of (200 μM and 1330 μM =) 0.2 and 1.33 mM employed in the *in vitro* studies (Table 2).

Concerning Glutaraldehyde, no specific PBK models have been published. Likely due to its high reactivity, also, no human biomonitoring data are available. This makes a quantitative judgement as to dosimetry complicated. Cable et al. (2025) performed, for many chemicals including GLUT, generic PBK modelling, to predict plasma C_max_. Assuming dermal exposure only, and considering both low and high exposure scenarios and employing different generic PK models, these authors predicted plasma Cmax in between 0.08 and 0.16 μM. (see Supplementary data to Cable et al. (2025), file “toxsci-24-0360-File009.xlsx,” tab “S2c. Cmax predictions_all”). The concentrations employed in the toxicogenomics studies considered here are 10 μM and 190 μM, around ∼2 to ∼3 orders of magnitude higher.

Concerning TDI, we modified our previous PBK model (Scholten et al., 2023) to calculate the concentration of TDI in interstitial fluid. We also estimated the concentration in lung epithelial lining fluid (see Supplementary Data File “S9_PBK_modelling_TDI.pdf”). Assuming an external exposure scenario of 40 μg/m^3^ for 4 h exposure, just exceeding the highest value reported in occupational studies (Figure 2A in Scholten et al. (2023), modelling predicted TDI concentrations of 0.023 µM in lung Interstitial fluid. An estimated concentration within the lung lining fluid (LLF) covering bronchial epithelial cells resulting from one inhalation was 0.575 x10^−3^ μM. Comparing these values to Mutz-3 dendritic cells (40 µM) and 16HBE14o epithelial cells (150 µM) (Table 2), these concentrations are around 1739 and several order of magnitude fold higher, respectively.

Hansen et al. (Hansen et al., 2014) reported MA-induced occupational asthma, together with the presence of MA-specific IgE antibodies in serum, indicating that internal exposure is directly related to MA-induced asthma. However, no quantitative exposure information in terms of tissue or blood concentrations, etc., has been reported in the literature, likely due to the high reactivity of MA. Further, searching PubMed, no reported PBK models are available for MA.

For TMA, another highly reactive chemical, neither biomonitoring data nor detailed PBK models are available from literature. Cable et al. (2025) provided various plasma C_max_ using generic PBK modelling. Considering inhalation exposure, at low occupational exposure limit value, C_max_ was predicted to range from ∼0.009 μM to ∼0.01 μM. At higher exposure scenarios, from 0.17 μM to 0.19 μM (see Supplementary Data to Cable et al. (2025), file “toxsci-24-0360-File009.xlsx”, tab “S2c. Cmax predictions_all”). The concentrations used in the toxicogenomics studies (150 μM and 2730 μM, Table 2) exceed these predicted concentrations (Table 2).

## Discussion

Establishing the relation between unique external exposures within the occupational exposome, such as chemical sensitisers and irritants, and health effects is challenging. Here, we hypothesised that human mechanistic information could be informative to generate hypotheses, supporting the plausibility of relationships between exposure and related health effects. We explored a data-driven mechanistic approach, grounded in human biology, based upon overlap in GO BPs derived from AOPs and disease-associated genes, and human toxicogenomics data. We examined GO BP overlap between irritants and LFD, and sensitisers and AA, respectively. Further, we considered the *in vitro* concentrations employed, in comparison to equivalent internal exposures expected from relevant external occupational exposure scenarios, inferred from PBK modelling and biomonitoring data.

Considering LFD, we observed overlap in GO BPs, with toxicogenomics data obtained from UFP exposed lung epithelial cells (Figure 4, Supplementary Data File S6). Interestingly and consistently, the regulation of GO BPs common to different timepoints were observed, including negative (GO:0050866, 1h) or positive (GO:0050867, 20h) regulation of cell activation, positive regulation of ion transport (GO:0043270: 4h, 20h) and stem cell proliferation (GO:0072089: 8h,20h). Further, at 20 h of exposure, the overlap between exposure and disease associated GO BPs was the highest (9 processes). Thus, expert curated GO BP content was usefull to capture persistent mechanistic signals from *in vitro* data, potentially relevant to understand the development of LFD. A more detailed evaluation from these GO BPs suggests the following mechanistic interpretation. UFPs are known to interact with transmembrane receptors and ion channel regulators, induce oxidative stress and inflammatory responses (innate and adaptive) in human and rodent models (Leikauf et al., 2020; Deiuliis et al., 2012). Studies have indicated that UFP/DEP exposure can alter TLR signalling, ion channels (e.g., TRPA1, TRPV4) (Shoenfelt et al., 2009; Schwarze et al., 2013; Li et al., 2011). While our approach lacks complex cell-cell interaction, it is suggested from the overlapping GO BPs (GO:0050867, GO:0043270) that these processes may be activated. UFPs induce oxidative stress and alter mitochondrial activity, induce cell membrane damage, stimulate lung dendritic cells and alter cell proliferation (Vallabani et al., 2023; de Haar et al., 2008; Lambrecht and Hammad, 2010; Leikauf et al., 2020; Provoost et al., 2010; Sydlik et al., 2006), suggested from GO BPs, including response to oxidative stress (GO:0006979), T cell activation (GO:0042110). The results may also indicate UFP induced compensatory proliferation through enrichment of different GO BPs (GO:0050673, GO:0048144, GO:0070661, GO:0072089). Together, a consistent, sustained time-dependent activation of human disease GO BPs was observed in human *in vitro* toxicogenomics studies, of possible relevance to understand the development of LFD.

Next, we identified GO BPs relevant to the development of AA. However, only a very small overlap was observed between expert curated GO BPs, and GO BPs enriched in 16HBE14o cells (epithelial uptake) and MUTZ-3 cells (dendritic cell migration) upon *in vitro* exposure to sensitisers (Figures 5, 7, Supplementary Data File S7, S8). Assuming that these models are capable of representing at least some of the different stages in the development of AA after sensitisation from occupational chemicals (as classification models using these cells have shown correct classification of sensitisers from irritants (Vandebriel et al., 2010), an assumption can be that if no GO BPs are activated, no sensitisation occurs. This assumption is based on the premise that the activation of specific BPs is crucial for the sensitisation process, and without such activation, the cascade of events leading to allergic asthma would not be initiated. However, individual MIE and KE of AOPs may comprise dozen or even hundreds of individual biochemical and molecular events (e.g., inflammation, oxidative stress). So, during the process of curating our key mechanisms, large numbers of events are binned, facilitating a simplistic mechanistic overview of an ultimate AOP for AA. Also, although we followed a strict set of inclusion and exclusion criteria (see methods “Retrieval of GO Biological processes, general considerations and steps”) a certain amount of selection bias can not be fully excluded. Moreover, as AOPs are linear and unidirectional, these do not contain compensatory mechanisms and are primarily limited to the sensitisation phase of asthma only and likely do not yet capture the full elicitation phase (e.g., involving mast cells, basophils, eosinophils). Therefore, the retrieved expert information on MIEs and KEs for AA as described above, is probably incomplete. This may, next to *in vitro* study shortcomings (discussed later), explain the limited overlap we observed in GO BPs. To augment the analysis, we therefore included GO BPs derived from DisGeNET -a gene-disease centered database constructed from expert curation and unbiased textmining of scientific literature-into the AA analyses, and indeed observed a much higher overlap (Jaccard indices were about one order of magnitude (∼10-fold) higher (compare Figure 6 with Figures 5, 8 with Figure 7 respectively)). As for UFP and LFD, this enabled a more detailed examination of the overlapping GO BPs (S7_Dik_overlap_AA.xlsx; and S8_Forreryd_overlap_AA.xlsx (tab PA; column N (“Overlap”)) towards a possible mechanistic interpretation, outlined below for PA as example. When PA appears to affect epithelial cell activation and downregulate cellular junction organisation, the integrity of the airway epithelial barrier may be compromised. This increased permeability may allows allergens and environmental triggers to penetrate more easily, exacerbating asthma symptoms. Simultaneously, the suggested upregulation of ROS metabolic processes results in enhanced production of reactive oxygen species (ROS), causing oxidative stress. This oxidative stress may damage cellular components, augment inflammatory responses, and contribute to airway hyperresponsiveness and mucus hypersecretion. Activated granulocytes, influenced by PA, release a plethora of inflammatory mediators, including cytokines and chemokines. This could amplify the inflammatory milieu and contributes to airway remodeling. Additionally, alterations in anion transport may affect the regulation of airway surface liquid, leading to changes in mucus viscosity, increased airway hyperresponsiveness, and potential modifications in the pH of the airway surface liquid. Furthermore, the suggested upregulation of the Type 2 immune response may lead to increased production of cytokines such as IL-4, IL-5, and IL-13. These cytokines promote eosinophilic inflammation, mucus production, and IgE synthesis, exacerbating asthma symptoms. The increased response to IL-1, a pro-inflammatory cytokine, may enhance the inflammatory response, leading to increased recruitment of immune cells to the airways and further promoting inflammation and tissue damage. Enhanced leukocyte migration results in the accumulation of various immune cells, including eosinophils, neutrophils, and lymphocytes, in the airways. This contributes to chronic inflammation and airway hyperresponsiveness. Altered regulation of growth factors leads to changes in airway structure, including increased smooth muscle mass and fibrosis, contributing to airway remodeling and persistent airflow obstruction. Increased chemotaxis enhances the movement of immune cells towards the site of inflammation, further amplifying the inflammatory response and contributing to tissue damage and airway remodeling. As a consequence of chronic inflammation, there is increased proliferation of airway smooth muscle cells, leading to thickening of the airway walls and increased airway resistance, which are characteristic features of asthma. Collectively, the GO BP overlap data may indicate that PA triggers various processes, and these processes perpetuate chronic airway inflammation, hyperresponsiveness, and tissue remodeling, establishing a cycle of inflammation and structural changes in the airways that manifest as the characteristic symptoms of asthma.

Aside mechanistic inference of GO BP based exposure disease associations, we also addressed a secondary hypothesis to identify if commonality exist in exposure-related activation of GO BPs reflecting diseases mechanisms *across different chemicals*. We considered the Dik et al. (2015). Indeed, for AA in combination with sensitisers, we demonstrate overlap in 20 GO BPs common to all 5 sensitisers tested *in vitro* (Supplementary Data File S7 (sheet tab “overlapping GOs all chem”). Together, this “GO BP signature” may be further developed in future as representative for the exposure to sensitisers within the occupational exposome.

We are aware of possible artefacts in our approach and a discussion on these is warranted.

First, shortcomings exist in the *in vitro* models considered here. Although plausible mechanistic interpretations could be provided for UFP and PA, the latter with DisGeNet derived GO BPs, capturing the interplay between different immunological cell types (e.g., release of cytokines and chemokines) is incomplete, as the studies were limited to bronchio epithelial (Grilli et al., 2018; Dik et al., 2015) and dendritic cells (Forreryd et al., 2015). Further, differences exist in time windows between short term *in vitro* exposure and toxicogenomics analysis, *versus* long term latency *in vivo* between exposure and disease outcome. This may also involve the existence of possible (mechanistic) feedback loops absent *in vitro*. As such, the *in vitro* models identified likely do not “capture the full AOP content”, and its time and concentration-dependent chemical activation, as would be expected to occur in multiple target cells in humans *in vivo*.

Second, *in vitro* to *in vivo* differences in chemical absorption, distribution, metabolism, excretion, and consequently dosimetry, exist. These differences need to be characterised to define the relevance of dose dependent toxicological effects *in vitro* for understanding *in vivo* effects. For the majority of sensitisers, using PBK modelling or published data, we were able to compare internal concentrations predicted/estimated from realistic occupational exposure scenarios with *in vitro* concentrations (Table 2)). For 3 out of the 5 chemicals (GLUT, TMA and TDI), *in vitro* concentrations were much higher in comparison to internal concentrations estimated from *in vivo* exposures. However, as (low) dose response data were absent in the *in vitro* studies, it is not clear if in target cells in humans exposed *in vivo*, similar gene expression/GO BPs changes would occur. Also, for GLUT and TMA, only plasma C_max_ values were predicted using generic PBK modelling under the assumption of only dermal exposure for GLUT and inhalation exposure for TMA (Cable et al., 2025). For TDI, PBK modelling could be performed upon inhalation exposure to calculate concentrations in lung interstitial fluid, for comparison with concentrations used in dendritic cells (Forreryd et al., 2015). For comparison with bronchio epithelial cells (Dik et al., 2015), data was lacking and only an approximate lung epithelial lining fluid concentration estimate (assuming one inhalation only) could be provided. For PA, the internal concentrations projected from urinary biomonitoring data (Pfaffli, 1986) were much higher in comparison to concentrations employed *in vitro*, at which GO BP (primarily from DisGeNet) overlap was observed. Given these estimates were in the upper millimolar range, an overestimation can not be excluded. Aside, as details of ADME for PA are unknown, it is unknown how urinary concentrations would compare to concentrations in lung tissue. Together, *in vitro* to *in vivo* dosimetry comparisons are complicated by incomplete PBK models, ignorance of relevant exposure routes for model input, uncertainty as to the relevance of using concentrations in organs/biofluids other than lung tissues (urine, plasma C_max_ *versus* interstitial), as well (as absence of or) diverging concentration ranges between *in vitro* and *in vivo*. Finally, the reactivity of these sensitizing chemicals may further complicate PBK and biomonitoring inferred dosimetry comparisons. This reactivity may also lead to an underestimation of the actual concentration *in vitro*. The nominal concentration *in vitro* may not be equal to the actual concentration reaching cells, due to binding to proteins in cell culture media supplements such as serum or albumin.

Third, the issue of available toxicogenomics data and AOPs needs to be discussed, as this affected the GO BP overlap analysis outcome. At the time of our study, OECD-endorsed AOPs - which undergo rigorous international peer review and offer a standardized, globally vetted framework -for AA and LFD were not available. The number of relevant AOPs (or pathways of toxicity) is expected to be limited by design (Kleensang et al., 2014), this due to the limited number of cellular targets and metabolic routes in human biology. However, it is inherently difficult to define what constitutes a “sufficient, true” number of AOPs and with this GO BPs to fully capture the chemically-induced disease processes. Further, although stringent selection criteria were applied to select AOP content and GO BPs, some bias can not be excluded. The restricted number of toxicogenomics studies retrieved was the result of applying stringent selection criteria as well, to ensure a.o. biological relevance, consistency in chemical testing and availability of full genome expression data in order to ensure GO BP coverage. While this approach enhances both chemical and biological mechanistic specificity, we realize that it also narrows the dataset pool. In future, our approach could be improved once standardized metadata and ontologies and FAIR data principles (Wilkinson et al., 2016) in toxicology are further implemented to facilitate findability and reuse of data. Further, maturation of OECD-endorsed AOPs, further integration of AI and NLP tools and similar approaches (e.g., AOP Helpfinder 3.0 (Jaylet et al., 2024), together with community-driven curation platforms, similar to AOP-Wiki, can improve transparency.

Fourth, AOPs documented under the AOP wiki are generally textual descriptions of biological processes. The actual molecular annotation (e.g., including identifiers for genes/proteins/protein subunits involved in MIE and KEs, which can be used for cross-referencing to other databases) is frequently lacking or at best limited. Further, unclarity may exist if the MIE and KEs involve interaction with a transcript, with protein expression and/or modification and/or function of the protein. Consequently, the proper translation into GO BPs may be subject to error.

Finally, our approach is based upon generic AOPs and *in vitro* cells from one source/individual. It does not account yet for interindividual differences. Genetic predisposition and preexisting comorbidities, such as atopic dermatitis can lead to a.o. increased dermal permeability and immune priming resulting in increased susceptibility in the response to environmental agents.

In conclusion, we presented a data integration approach based upon curation and integration of AOP and disease data resources, together with well controlled mechanistic *in vitro* exposure to effect data. Although limitations in our approach exist (coverage of AOP content, uncertainty in dosimetry issues, isolated *in vitro* systems, lack of consideration of interindividual differences), our human-centric approach may contribute to the identification of plausible mechanistic links between exposures commonly present in the occupational exposome and disease outcomes. It can thereby complement and inform findings obtained from animal studies and epidemiology. Also, it represents a useful resource for further development of adverse outcome pathways, as we curated mechanisms (GO BPs) potentially associated with LFD and AA, with gene and GO BP content. Finally, the approach can identify commonality between different chemicals in terms of activating disease mechanisms, which may ultimately help to prioritise chemicals within the occupational exposome towards risk assessment.

## Data Availability

The original contributions presented in the study are included in the article/Supplementary Material, further inquiries can be directed to the corresponding author.

## Footnotes

1
https://www.ephor-project.eu/

2
https://disgenet.com/

3
https://aopwiki.org/

4
https://wikikaptis.lhasacloud.org/#/aop

5
https://www.genecards.org/

6
https://www.ebi.ac.uk/QuickGO/

7
https://pubmed.ncbi.nlm.nih.gov/

8
https://aopwiki.org/aops/39

9
https://aopwiki.org/aops/259

10
https://aopwiki.org/aops/40

11
https://geneontology.org/

12
https://pantherdb.org/

13
https://www.ebi.ac.uk/biostudies/diXa/studies

14
https://www.toxicodb.ca/

15
https://www.ncbi.nlm.nih.gov/geo/

16
https://www.ebi.ac.uk/biostudies/arrayexpress/studies/E-MTAB-5157

17
https://www.webgestalt.org/

18
https://echa.europa.eu/nl/information-on-chemicals

19
https://www.r-project.org/

20
https://www.ebi.ac.uk/biostudies/arrayexpress/studies/E-MEXP-3773?query=E-MEXP-3773

21
https://geneontology.org/stats.html

22
https://www.dsmz.de/collection/catalogue/details/culture/ACC-295

23 based upon the following calculation: The average concentration of creatinine can be estimated as 8.2 mmol/mL. (*The average urinary creatinine (24-h urine collection) values can be estimated at 11500 mmol/day, based upon a of 4420–17680 mmol/day. (https://www.mountsinai.org/health-library/tests/creatinine-urine-test). The average urine production is 1400 mL/day, based upon a normal range for 24-h urine volume of 800 to 2,000 mL per day (https://www.mountsinai.org/health-library/tests/urine-. Together, the average concentration of creatinine would equal 11500 mmol/1400 mL = 8.2 mmol/mL) Therefore, the average concentration range of PA from (Pfaffli, 1986), expressed as mmol/mL urine would be: 0.3–14 μmol/mmol creatine * 8.2 mmol creatinine/mL urine = 2.46–114.8 μmol/mL = 2.46–114.8 mM.
