# Supplementary material for: Occupational exposure and its mechanistic link to allergic asthma and lung function decline; a data-driven approach coupled to mining of adverse outcome pathway signatures
Source: Front Toxicol. 2025 Sep 8;7:1589380. doi: 10.3389/ftox.2025.1589380 (PMC12450882; doi:10.3389/ftox.2025.1589380)
Supplement: Supplementary file 4 [file Supplementaryfile1.pdf]

**PBK modelling to estimate the interstitial, and epithelial, concentrations after respiratory exposure to TDI.**

***Other Supplementary File 1.pdf to: Stierum et al.. Occupational exposure and its mechanistic link to allergic asthma and lung function decline; a data-driven approach coupled to mining of adverse outcome pathway signatures.***

We use a modification of our existing PBK model to model internal exposure upon work-related diiscocyanate exposure (Scholten, Westerhout et al. 2023).

We modified the model in order to predict the amounts/concentrations in the lung interstitial space, as a relevant compartment to model local exposure to (dendritic) cells residing in the interstitial space. Further, we assumed, next to the availability of free, unbound TDI ("unb"), the presence of TDI bound to albumin ("alb") and other macromolecules ("mcr") (Figure 1), and as described previously in paragraph 2.1.2 in Scholten et al. (Scholten, Westerhout et al. 2023).

First, in order to calculate the local concentration in the interstitial space, we retrieved information to estimate the volume of the lung interstitial space. The assumption here is that the lung interstitial space has a total surface area  of 85 $m^2$, based upon Ananda Rao (Ananda Rao, Johncy 2022). Secondly, the average cell thickness was assumed to be 2 μm (Crapo, Barry et al. 1982) (based upon an approximate average estimated from Table 2 from this publication: 0.35 μm for alveolar type 1, 5.02 μm for alveolar type 2, and 0.48 μm for endothelial cells). The total volume of interstitial space was then calculated as: 85 $m^2$ x 2 μm =  0.17 l (170 ml).

MacRea (MacRae, Joseph et al. 2006) mentioned that lung water volume water, the water content of the lung interstitium, equals 2 ml/kg body weight for the normal population. Assuming that all the volume of the interstitial fluid equals the total volume of lung water (as it mainly consists of water and electrolytes (Guyton, Hall 2006)), and an average body weight of 85 kg (as used within our previous publication (Scholten, Westerhout et al. 2023) , also a volume of 2 x 85 = 170 ml is estimated.

Figure 1 provides an overview of the PBK model.

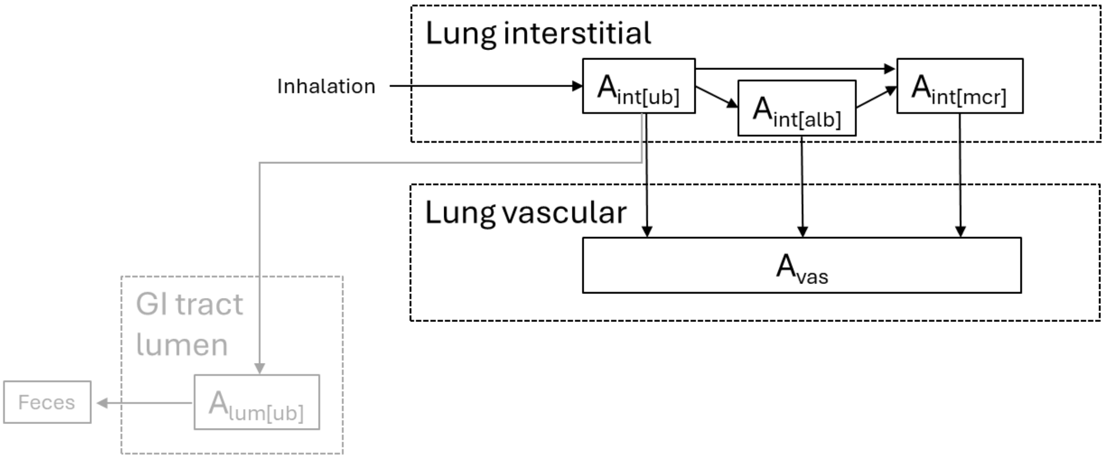


**Figure 1.** Overview of PBK model to estimate lung interstitial concentrations of TDI.

The input 'Inhalation' is the concentration in air (Cair, converted to µmol/L), multiplied by the absorbed fraction (Fabs, at 0.2) and the breathing rate (Qbr, L/h, at 1000 L/h) (from (Scholten, Westerhout et al. 2023). The three arrows within the "lung interstitial" compartment pointing downwards represent the uptake into the lung 'vascular total' compartment for mass balance. The light grey arrow represents the uptake by the gastrointestinal lumen (as represented in our original model), via the interstitial fluid, but can be ignored here from the mass balance since Fabs determines how much ends up in the lungs. The R-script representing the model is more detail available at the end of this file (page 6).

Next, we used a realistic exposure scenario, based upon exposure data from various occupational studies, as summarized in Figure 2A in Scholten et al. (Scholten, Westerhout et al. 2023). The scenario considered here was based upon an exposure of 40 µg/m$^3$ over 4 hours,  representing a value slightly exceeding the highest value reported in occupational studies. Using this exposure value, the model predicted a total amount in lung interstitial space of 0.0055 µmol for the sum of unbound + bound to albumin and other macromolecules (Figure 2).

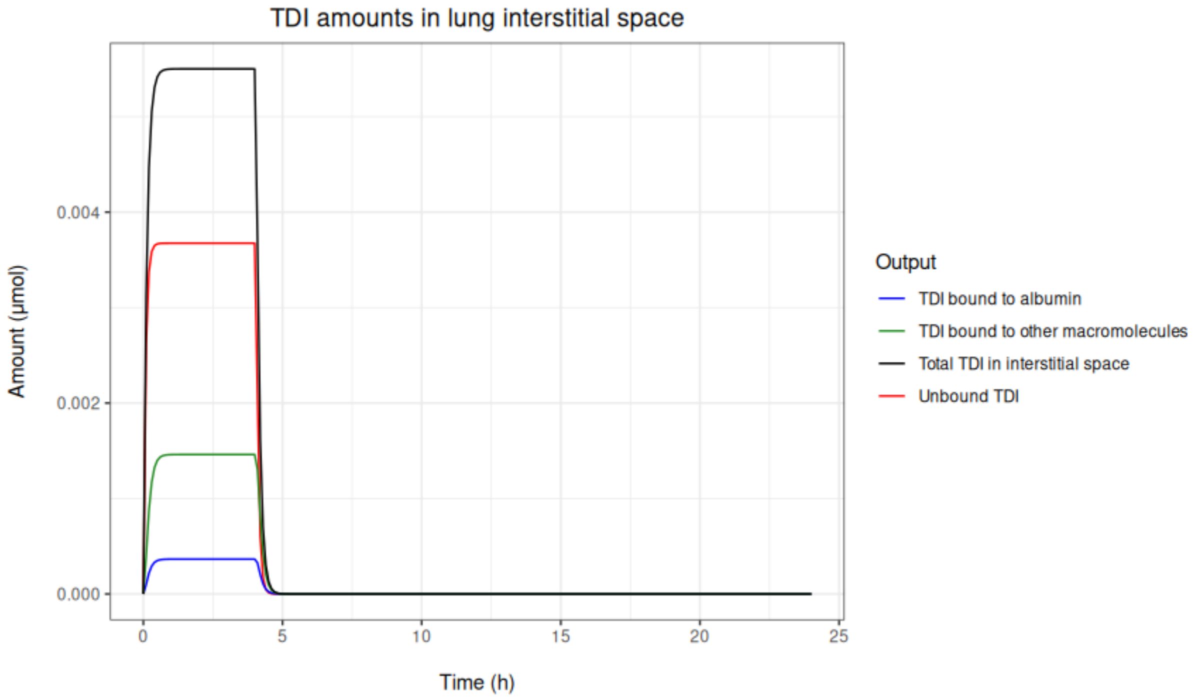


**Figure 2.** Predicted amounts of TDI within the lung interstitial space,external exposure scenario of 40 µg/m$^3$ over 4 hours as input. Next to the total amount estimated, the amounts are indicated for each of the subcompartiments (unbound, bound to albumin, bound to other macromolecules)

Assuming an interstitial volume of 170 mL, which seems realistic given that two approaches yielded the same value, the model predicted a total concentration in the lung interstitial space of 0.032 µM (0.0055 µmol/0.17 L = 0.032 µM) (Figure 3).

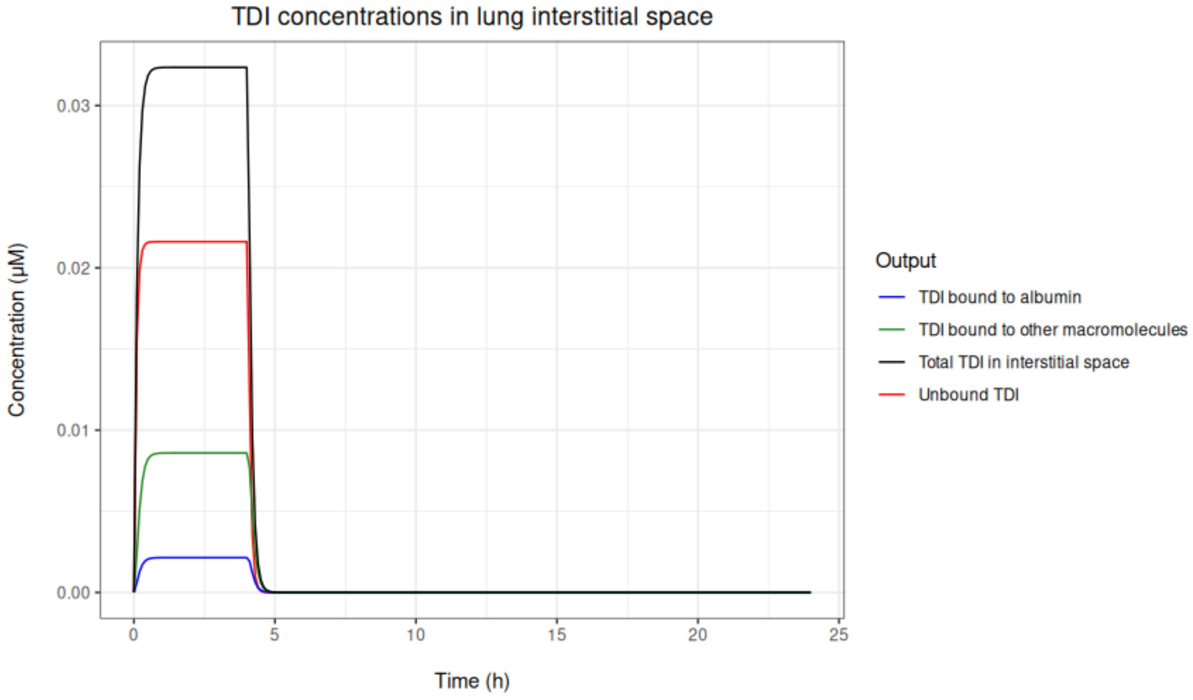


**Figure 3.** Predicted concentrations of TDI within the lung interstitial space, using an external exposure scenario of 40 µg/m$^3$ over 4 hours as input. Next to the total amount estimated, the concentrations are indicated for each of the subcompartiments (unbound, bound to albumin, bound to other macromolecules)

Thus, the used concentration of 40 µM (Table 2 of the Manuscript, 40 µM (Forreryd, Johansson et al. 2015) is more than 1000-fold higher compared to the predicted concentration of 0.032 µM. Conversely, to reach the same concentrations in dendritic cells as used in the vitro studies considered in our study, the external exposure would need to be more than 1000 times higher, in comparison to the external exposure concentration of 40 µg/m$^3$ expected in (the higher end of) realistic occupational exposure scenarios (likely not realistic).

Estimating the local concentrations on the apical side of the lung epithelium, in the lung lining fluid (LLF), to further aim for the comparison with the in vitro concentration of 150 µM used with the human bronchial epithelial cell line 16HBE14o (Dik, Pennings et al. 2015)) is more complicated, as we could not parametrize the PBK model for this purpose. Again an external exposure scenario of 40 µg/m$^3$ was assumed. Corrected for Fabs of 0.2, the actual air concentration at the epithelial cell lining would equal 8 µg/m$^3$. This corresponds to a concentration of 0.000046 µmol/L (air!). However, an air concentration of 0.000046 µmol/L is not directly comparable to the concentration applied in in vitro cell medium. If we assume an equilibrium between external exposure and the LLF, and between LLF and interstitial fluid, one inhaled breath (approximately 500 ml) would yield a bolus exposure of 0.000023 µmol (0.000046/2). To calculate the actual concentration at the epithelial cell layer, an estimate of the total volume of the lung epithelial lining fluid (LLF) is necessary. As reviewed and described in Fröhlich et al. (Fröhlich, Mercuri et al. 2016), determination of the volume of LLF is complicated, as no optimal method is available. Estimates range from 10ml - 70 ml (Fröhlich, Mercuri et al. 2016), with a calculated average here of (10 + 70)/2 = 40 ml in total for humans. Although the LLF was not a compartment within our PBK model, based upon this estimate, an average concentration at the epithelial lining resulting from one inhalation would be 0.000023 µmol/40 ml= 0.000000575 µmol/ml = 0.575 x10$^{-3}$ µM. Compared to the reported concentration in Dik et al. of 150 µM, this is several orders of magnitude lower (260869 fold).

Together, the TDI concentrations employed in both in vitro studies are much higher in comparison to the concentrations predicted (or estimated) in relevant compartments upon an external exposure scenario slightly exceeding the highest exposure reported in occupational studies.

**REFERENCES**

ANANDA RAO, A. and JOHNCY, S., 2022. Tennis Courts in the Human Body: A Review of the Misleading Metaphor in Medical Literature. *Cureus,* **14**(1), pp. e21474.

CRAPO, J.D., BARRY, B.E., GEHR, P., BACHOFEN, M. and WEIBEL, E.R., 1982. Cell number and cell characteristics of the normal human lung. *The American Review of Respiratory Disease,* **125**(6), pp. 740–745.

DIK, S., PENNINGS, J.L.A., VAN LOVEREN, H. and EZENDAM, J., 2015. Development of an in vitro test to identify respiratory sensitizers in bronchial epithelial cells using gene expression profiling. *Toxicology in vitro : an international journal published in association with BIBRA,* **30**(1 Pt B), pp. 274–280.

FORRERYD, A., JOHANSSON, H., ALBREKT, A., BORREBAECK, C.A.K. and LINDSTEDT, M., 2015. Prediction of chemical respiratory sensitizers using GARD, a novel in vitro assay based on a genomic biomarker signature. *PloS one,* **10**(3), pp. e0118808.

FRÖHLICH, E., MERCURI, A., WU, S. and SALAR-BEHZADI, S., 2016. Measurements of Deposition, Lung Surface Area and Lung Fluid for Simulation of Inhaled Compounds. *Frontiers in Pharmacology,* **7**.

GUYTON, A.C. and HALL, J.E., 2006. *Textbook of medical physiology.* 11th edn. Philadelphia: Elsevier Saunders.

MACRAE, J.M., JOSEPH, G., KISLUKHIN, V., KRIVITSKI, N.M., HEIDENHEIM, A.P. and LINDSAY, R.M., 2006. Determining Lung Water Volume in Stable Hemodialysis Patients: *ASAIO Journal,* **52**(4), pp. 430–437.

SCHOLTEN, B., WESTERHOUT, J., PRONK, A., STIERUM, R., VLAANDEREN, J., VERMEULEN, R., JONES, K., SANTONEN, T. and PORTENGEN, L., 2023. A physiologically-based kinetic (PBK) model for work-related diisocyanate exposure: Relevance for the design and reporting of biomonitoring studies. *Environment International,* **174**, pp. 107917.

# R-SCRIPT

```
# Simple TDI model

rm(list=ls()) # to clear out the global environment


# Set working directory

setwd("/home/westerj")


library(dplyr)

library(deSolve)

library(truncnorm)

library(reshape2)

library(ggplot2)

# library(nlme)

# library(minpack.lm)


Lung_interstitial <- function(t, A, parms) {

  with(as.list(c(A, parms)), {

    Cair = varCair(t)


    dAint_ub <- Qbr*Fabs*Cair - Kalb*Aint_ub - Kmcr*Aint_ub - Kvas*Aint_ub

    Cint_ub <- Aint_ub/Vint

    dAint_alb <- Kalb*Aint_ub - Kelim*Aint_alb - Kvas*Aint_alb

    Cint_alb <- Aint_alb/Vint

    dAint_mcr <- Kmcr*Aint_ub + Kelim*Aint_alb - Kvas*Aint_mcr

    Cint_mcr <- Aint_mcr/Vint

    dAvas_total <- Kvas*Aint_ub + Kvas*Aint_alb + Kvas*Aint_mcr


    Aint_total <- Aint_ub + Aint_alb + Aint_mcr

    Cint_total <- Aint_total/Vint


    list(c(dAint_ub,dAint_alb,dAint_mcr,dAvas_total),c(Aint_total=Aint_total, Cint_ub=Cint_ub,Cint_mcr=Cint_mcr,Cint_alb=Cint_alb,Cint_total=Cint_total)) # Important: this 'list' must contain all compartments in order to produce output

  })

}


#### PK parameters ----

time <- seq(0,24,by=0.1) # this creates a vector of a sequence from 0 to 24 in steps of 0.5


MW <- 174.16

Cair <- 40 # ug/m3

Cair_uM <- (40/1000)/MW

Qbr <- 1000 # L/h

Fabs <- 0.2 # -


exp_stop <- 4
```

```
Variables_df <- data.frame("time"=time,
                    "Cair"=Cair_uM)
Variables_df <- Variables_df %>%
  mutate(Cair = if_else(time > exp_stop,0,Cair)) # stop exposure after exposure_duration

varCair <- approxfun(Variables_df$time, Variables_df$Cair, rule = 2)

Falb <- 0.2
Talbmcr <- 0.167
Kalb <- Falb*log(2)/Talbmcr # Rate of binding to albumin
Kmcr <- (1-Falb)*(log(2)/Talbmcr)

Tvas <- 0.083
Kvas <- log(2)/Tvas

Telim <- 456
Kelim <- log(2)/Telim

BW <- 85
VFlun <- 0.015
Vlun <- VFlun*BW # Volume of lung interstitial compartment (L)

SAint <- 85 # m2 [https://pmc.ncbi.nlm.nih.gov/articles/PMC8863270/]
Thickness <- 0.000002 # m [https://www.researchgate.net/publication/16096311_Cell_number_and_cell_characteristics_of_the_normal_human_lung#:~:text=Abstract,37%25%20of%20the%20total%20cells.]
Vint <- SAint*Thickness*1000 # m3 -> L

A_init <- c(Aint_ub = 0,
          Aint_alb = 0,
          Aint_mcr = 0,
          Avas_total = 0)

parms <- c(#Cair=Cair,
         Fabs=Fabs,
         Qbr=Qbr,
         Kalb=Kalb,
         Kmcr=Kmcr,
         Kvas=Kvas,
         Kelim=Kelim,
         Vint=Vint)

output_TDI <- as.data.frame(lsoda(A_init, time, Lung_interstitial, parms))

#### Figures ----
Aint_plot <- ggplot() +
  geom_line(data = output_TDI, aes(x = time, y = Aint_ub, colour = "Aint_ub")) +
  geom_line(data = output_TDI, aes(x = time, y = Aint_alb, colour = "Aint_alb")) +
  geom_line(data = output_TDI, aes(x = time, y = Aint_mcr, colour = "Aint_mcr")) +
```

```
  geom_line(data = output_TDI, aes(x = time, y = Aint_total, colour = "Aint_total")) +

  scale_colour_manual(name='Output',
                      values=c('Aint_ub'='red',
                               'Aint_alb'='blue',
                               'Aint_mcr'='forestgreen',
                               'Aint_total'='black'),
                      labels=c('Aint_ub'='Unbound TDI',
                               'Aint_alb'='TDI bound to albumin',
                               'Aint_mcr'='TDI bound to other macromolecules',
                               'Aint_total'='Total TDI in interstitial space')) +

  ylab("Amount (\u03BCmol)\n") + xlab("\nTime (h)") + theme_bw() +
  ggtitle("TDI amounts in lung interstitial space") +
  theme(plot.title = element_text(hjust = 0.5))

Aint_plot

TDI_plot <- ggplot() +
  geom_line(data = output_TDI, aes(x = time, y = Aint_ub, colour = "Aint_ub")) +
  geom_line(data = output_TDI, aes(x = time, y = Aint_alb, colour = "Aint_alb")) +
  geom_line(data = output_TDI, aes(x = time, y = Aint_mcr, colour = "Aint_mcr")) +
  geom_line(data = output_TDI, aes(x = time, y = Aint_total, colour = "Aint_total")) +
  geom_line(data = output_TDI, aes(x = time, y = Avas_total, colour = "Avas_total")) +

  scale_colour_manual(name='Output',
                      values=c('Aint_ub'='red',
                               'Aint_alb'='blue',
                               'Aint_mcr'='forestgreen',
                               'Aint_total'='black',
                               'Avas_total'='orange'),
                      labels=c('Aint_ub'='Unbound TDI',
                               'Aint_alb'='TDI bound to albumin',
                               'Aint_mcr'='TDI bound to other macromolecules',
                               'Aint_total'='Total TDI in interstitial space',
                               'Avas_total'='Total TDI in lung vascular space')) +

  ylab("Amount (\u03BCmol)\n") + xlab("\nTime (h)") + theme_bw() +
  ggtitle("TDI amounts in lung interstitial space") +
  theme(plot.title = element_text(hjust = 0.5))

TDI_plot

Cint_plot <- ggplot() +
  geom_line(data = output_TDI, aes(x = time, y = Cint_ub, colour = "Cint_ub")) +
  geom_line(data = output_TDI, aes(x = time, y = Cint_alb, colour = "Cint_alb")) +
  geom_line(data = output_TDI, aes(x = time, y = Cint_mcr, colour = "Cint_mcr")) +
```

```
  geom_line(data = output_TDI, aes(x = time, y = Cint_total, colour = "Cint_total")) +

  scale_colour_manual(name='Output',
                      values=c('Cint_ub'='red',
                               'Cint_alb'='blue',
                               'Cint_mcr'='forestgreen',
                               'Cint_total'='black'),
                      labels=c('Cint_ub'='Unbound TDI',
                               'Cint_alb'='TDI bound to albumin',
                               'Cint_mcr'='TDI bound to other macromolecules',
                               'Cint_total'='Total TDI in interstitial space')) +

  ylab("Concentration (\u03BCM)\n") + xlab("\nTime (h)") + theme_bw() +
  ggtitle("TDI concentrations in lung interstitial space") +
  theme(plot.title = element_text(hjust = 0.5))

Cint_plot
```
